# Reliability and stability of tactile perception in the whisker somatosensory system

**DOI:** 10.3389/fnins.2024.1344758

**Published:** 2024-05-30

**Authors:** Hariom Sharma, Rony Azouz

**Affiliations:** Department of Physiology and Cell Biology, Zlotowski Center for Neuroscience, Ben-Gurion University of the Negev, Be’er Sheva, Israel

**Keywords:** somatosensory system, whiskers, textures, cortex, sensory processing. perceptual constancy

## Abstract

Rodents rely on their whiskers as vital sensory tools for tactile perception, enabling them to distinguish textures and shapes. Ensuring the reliability and constancy of tactile perception under varying stimulus conditions remains a fascinating and fundamental inquiry. This study explores the impact of stimulus configurations, including whisker movement velocity and object spatial proximity, on texture discrimination and stability in rats. To address this issue, we employed three distinct approaches for our investigation. Stimulus configurations notably affected tactile inputs, altering whisker vibration’s kinetic and kinematic aspects with consistent effects across various textures. Through a texture discrimination task, rats exhibited consistent discrimination performance irrespective of changes in stimulus configuration. However, alterations in stimulus configuration significantly affected the rats’ ability to maintain stability in texture perception. Additionally, we investigated the influence of stimulus configurations on cortical neuronal responses by manipulating them experimentally. Notably, cortical neurons demonstrated substantial and intricate changes in firing rates without compromising the ability to discriminate between textures. Nevertheless, these changes resulted in a reduction in texture neuronal response stability. Stimulating multiple whiskers led to improved neuronal texture discrimination and maintained coding stability. These findings emphasize the importance of considering numerous factors and their interactions when studying the impact of stimulus configuration on neuronal responses and behavior.

## Highlights

• Rats depend on their whiskers to sense their environment and distinguish textures and shapes.
• This research investigates how their ability to maintain a consistent sense of touch is affected by changing conditions.
• Variables such as whisker speed and object distance were examined.
• These changes influenced their tactile perception but didn’t hinder their ability to differentiate textures.
• However, keeping their touch sensation steady made it challenging for them.
• The study also explored how the rats’ cortical neurons responded to these changing conditions, causing instability in their touch perception.
• Using multiple whiskers improved their performance and stability.
• The study underscores the need to consider various factors when studying rat behavior and brain functions.

## Introduction

Perceptual constancy is a fundamental aspect of sensory perception that allows organisms to maintain a stable perception of objects, shapes, and textures despite variations in sensory inputs. The concept of perceptual constancy suggests that the nervous system considers the conditions affecting subject-object interactions and employs sensory processing mechanisms to correct for potentially distorted stimuli. Perceptual constancy has been extensively studied in the visual (Quiroga et al., 2005; Zoccolan et al., 2007; Rust and Dicarlo, 2010; DiCarlo et al., 2012; Sharpee et al., 2013), auditory (Bendor and Wang, 2005; Barbour, 2011; Bizley and Cohen, 2013; Rabinowitz et al., 2013), olfactory (Stopfer et al., 2003; Cleland et al., 2007), and somatosensory (DiCarlo and Johnson, 1999; Berryman et al., 2006; Pei et al., 2010; Yoshioka et al., 2011) systems. Experimental and theoretical studies have proposed several computational models, primarily focused on the visual system (Hubel and Wiesel, 1962; Maloney and Wandell, 1986; Craven and Foster, 1992; Brainard and Freeman, 1997; Brainard, 1998; Zaidi, 1998; Riesenhuber and Poggio, 1999; Amano et al., 2005; Kouh and Poggio, 2008) and primate somatosensory system (Pei and Bensmaia, 2014; Lieber and Bensmaia, 2020), aiming to explain how the brain achieves invariant stimulus representation. However, there is still a need for a more comprehensive understanding of the specific computations employed by the brain to generate consistent neural representations and achieve perceptual constancy.

Rodents heavily rely on their whiskers as specialized tactile sensors, enabling them to navigate and interact with their environment effectively (Carvell and Simons, 1990; Brecht et al., 1997; Harvey et al., 2001; Krupa et al., 2001; Kleinfeld et al., 2006; Diamond et al., 2008; Lottem and Azouz, 2009; Diamond, 2010; Jadhav and Feldman, 2010; Morita et al., 2011; Kuruppath et al., 2014). This system gives rodents precise information about object properties, texture discrimination, and spatial localization. However, sensory information obtained from whisker deflections is prone to fluctuations due to factors such as whisker movement velocity resulting from head movements and the proximity of objects. Perceptual constancy in touch, specifically in the perception of texture among primates, has been prominently observed. The sensing mode (receptive vs. active) has been demonstrated to influence the roughness perception. Receptive sensing, where the surface is moved, shows significant pressure and sensing velocity effects on the roughness perception. On the other hand, active sensing does not exhibit the same effects of velocity and force (Lederman, 1981; Yoshioka et al., 2011; Tanaka et al., 2014; Boundy-Singer et al., 2017).

The current study delves into how rodents ensure reliable and consistent tactile perception despite significant variations in stimulus conditions. This inquiry focuses on how animals compensate for changes in whisker movement speed and object spatial proximity, among other factors, to maintain a consistent and accurate perception of textures. Our research began by examining stimulus configurations’ influence on whisker-surface interactions. Building upon that, we examined how these configurations affected texture discrimination and stability in awake head-fixed rats. Finally, our exploration expanded to investigating the effects of stimulus configurations on cortical neuronal responses and their potential correlation with behavioral performance.

## Materials and methods

### Animals and surgery

Sprague Dawley rats from both sexes (250–320 gm) were anesthetized with ketamine (100 mg/kg, i.p.; Ketaset; Fort Dodge Animal Health, Fort Dodge, IA) and acepromazine maleate (1 mg/kg, i.p; PromAce; Fort Dodge Animal Health). After tracheotomy, a short (1.5 cm) metal cannula [outer diameter (o.d.), 2 mm; inner diameter (i.d.), 1.5 mm] was inserted into the trachea. The rats were then placed in a standard stereotaxic device. Body temperature was kept at 37.0 ± 0.1°C using a heating blanket and a rectal thermometer (TC-1000; CWE, Ardmore, PA). Anesthesia was maintained using a mixture of halothane (0.5–1.5%) and air employing artificial respiration at 100–115 breaths/min while monitoring end-tidal CO_2_ levels and heart rate. Depth of anesthesia was monitored based on heart rate (250–450 bpm), eyelid reflex, pinch withdrawal, and vibrissae movements. Halothane concentrations were set slightly above the level at which the first clear signs of vibrissae movements were observed while the eyelid reflex was still maintained. In some animals, we also used EEG recordings obtained using two wires inserted under the skull at a distance of 10 mm antero-caudally. Based on these measurements, we determined the anesthesia level in our recordings to be between stages III-2 and III-3 (Friedberg et al., 1999). After placing the subjects in a stereotactic apparatus (TSE, Bad Homburg, Germany), an opening (1–2 mm in diameter) was made above the barrel cortex (centered at 2.5 mm posterior and 5.2 mm lateral to the bregma), and the dura mater was carefully removed.

In some animals, we determined the correspondence between Microdrive depth and laminar identity. We induced electrolytic lesions using the recording electrodes by applying a direct current (10–30 μA) for 4 s, at a depth corresponding to each recorded area. In some rats, brain tissues were also processed for Cytochrome Oxidase histochemistry. The animals were perfused transcardially with 2.5% glutaraldehyde and 0.5% paraformaldehyde, followed by 5% sucrose, all in 0.1M PBS (Phosphate Buffered Saline). Brains from these rats were then transferred to a 30% sucrose post-fixative solution and incubated overnight at 4°C. The following day, microtome cryosections (120 μm) were prepared and incubated in PBS containing 0.0015% cytochrome C (Sigma) and 0.05% diaminobenzidine 20–50 min at 37°C. The reaction was terminated by washing with PBS. CO-stained sections were mounted on gelatin-coated slides, air-dried, and coverslipped. Layers 2/3, 4, 5, and 6 were identified by recording depths of 150–550, 550–850, 900–1400, and 1400 μm and deeper, respectively.

### Behavioral training and surgery

The rats were trained to discriminate between textures and the same texture, placed at different distances, and moved at different velocities. We employed the experimental paradigm devised by Waiblinger et al. (2015). Head mounts are implanted in a stereotactic apparatus under anesthesia Ketamine–Domitor (80 and 0.5 mg/kg, SC) and treated with Rimadyl (5 mg/kg, SC in 1 ml Ringer’s solution). Briefly, the rat’s head fur was shaved, and the skin was disinfected. Lidocaine cream (2%) was applied to the ears, and the rat was mounted in a stereotaxic device. Lidocaine (5%) was injected under the scalp along the midline. A 2–2.5 cm midline incision was made on the scalp, exposing the skull. Holes were drilled in the head for stainless steel screws. The screws were placed symmetrically in specific locations. Exposed bone and screws were covered with a thin layer of 4-META resin cement. Sterile sutures close the skin. Antibiotics and analgesics were administered, and the rats recovered in a cage with a heating pad. Post-operative care included antibiotics, analgesics, and access to food and water. Post-operative care included the administration of antibiotics (Pen-Strep, 2 ml/kg SC), analgesics (Rimadyl; 5 mg/kg SC), and ad libitum food and water.

Behavioral training commenced 1 week after surgery. Rats were habituated to the experimental situation by subjecting them to a systematic desensitization procedure for 2–3 weeks, after which all animals tolerated head fixation without any sign of stress. They are then placed on water restriction, and conditioning commences.

Rats underwent training on a psychophysical task known as the detection of change (DOC). Inspired by a study conducted by Waiblinger et al. (2015). We utilized a modified version of the Go/NoGo task. The rats were trained to perform a licking response when they detected a change in the coarseness of a surface. Each trial began with a 1 kHz sound cue. A wheel covered with textures was then brought within reach of the C3 whisker, taking approximately 0.2 s to reach the designated position. The wheel started rotating immediately at 147.3 degrees per second, lasting about 1 s. In the first experimental paradigm, the wheel was covered with two different textures, exposing the rats to both surfaces for about 1 s. Following this, the wheel was retracted out of reach of the whisker.

The rats were trained to lick only after receiving a specific cue, which was a sound signal of 5 kHz. This cue occurred 0.5 s after the wheel started moving and 0.5 s after the wheel was retracted. If the rats licked outside this specific period, the trial would end, and they would experience a brief timeout of 2 s before the subsequent trial began. During this task, the rats had to detect a difference between two stimuli labeled S^+^ and S^–^ and respond by licking the lickport only when they sensed that difference. Upon correctly licking the lickport after the go cue, the rat received a small drop of liquid reward consisting of 0.3% sucrose in water.

In the first paradigm, we consistently designated the rough surface (P120) as S^+^ and initially used the smooth surface (S^–^) as the starting stimulus during training. Once the rats learned this task, we modified the S^–^ stimulus using P800, P400, P220, and P120 as alternative stimuli. Each daily session consisted of 75–150 trials to ensure the rats received adequate water. Among these trials, 50–75% presented a more challenging task, while the remaining trials involved the detection of the P120-smooth stimulus. The different stimuli were presented in a pseudorandom order.

In the second paradigm, each trial began with a 1kHz sound cue. Initially positioned out of reach, the wheel covered with a surface texture was moved towards the C3 whisker. This movement of the wheel into the reachable plane took approximately 0.2 s. At the same time, the wheel immediately started to rotate, reaching its desired speed in about 0.2 s. In this paradigm, the wheel was covered with either the P120 or P400 texture and rotated at a speed of 147.3 deg/sec. As depicted in Figure 2E (lower panel), the surface interacted with the whisker tip. The wheel continued rotating for approximately 0.7 s before retracting. After the retraction, the wheel moved back into contact with the whisker, either at a different location on the wheel (designated as S^–^; Smooth, P400) or at the exact location but positioned at a different distance from the whisker tip (10 mm away), or a different velocity (169.5 degrees per second). The rats were allowed to lick only after receiving the go cue, a 5kHz sound that occurred 0.5 s after the start of the wheel’s second movement and 0.5 s after the wheel retraction.

All experiments were conducted following appropriate international standards and were approved by the Ben-Gurion University (BGU) Committee for the Ethical Care and Use of Animals in Research (project license: IL-71-11-2016). The BGU animal care and use program is supervised and fully assured by the Israeli Council for Animal Experimentation of the Ministry of Health. It is operated according to Israel’s Animal Welfare Act of 1994 and follows the Guide for Care and Use of Laboratory Animals (NRC 2011). In addition, BGU is approved by the Office of Laboratory Animal Welfare, USA (OWLA) (#A5060-01). Sprague Dawley rats from both sexes (200–300 g) were used for all experiments described herein.

### Recording technique

A multi-contact silicone electrode (NeuroNexus, Ann Arbor, Michigan) was inserted into the barrel cortex. The electrode was lowered using a precision stereotactic micromanipulator (TSE-systems, Germany). During recording, signals were amplified (1000x), digitized (25 kHz), filtered (0.1–10,000 kHz), and stored for offline spike sorting and analysis using the ME-16 amplifier and MC-Rack software (MEA, Germany). Data were then separated into local field potentials (LFP; 1–150 Hz) and isolated single-unit activity (0.5–10 kHz). All neurons could be driven by the manual stimulation of one of the whiskers. Spike extraction and sorting were implemented using the MClust (by A.D. Redish)^1^ MATLAB (Mathworks, Natick, MA)-based spike-sorting software. The extracted and sorted spikes were stored at a 100 μs resolution, and peri-stimulus time histograms (PSTHs) were computed.

### Whisker stimulation and recording

**FIGURE 1:**
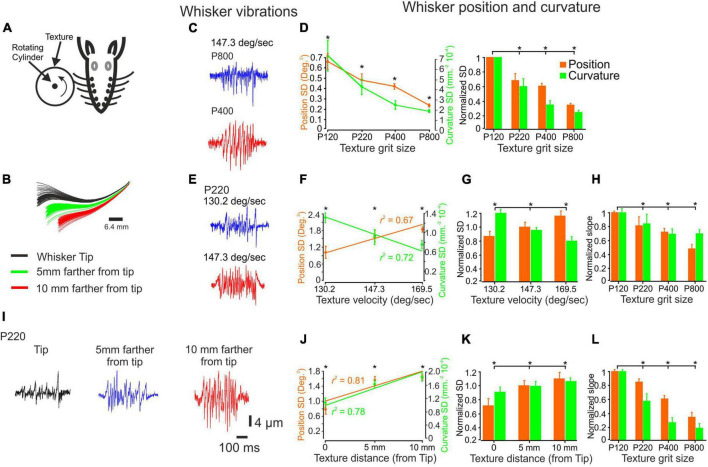
The influence of changes in surface coarseness and stimulus configurations on whisker vibrations. **(A)** Experimental design. The whiskers are contacting a rotating cylinder covered with textured sandpaper. **(B)** An example showcasing the influence of surface distance is demonstrated through several frames. **(C)** The panel provides a visual representation of the vibrations exhibited by a C3 whisker when encountering two textures (P400, P800) as a wheel touches the tip of the whisker, moving at a velocity of 147.3 deg/sec. **(D)** The influence of texture coarseness on the SD of whisker vibration position (orange) and curvature (green) for the whiskers in **(C)** (left panel). Asterisks indicate statistically significant differences between the groups (*p* < 0.01). The impact of texture coarseness was examined across all recorded whiskers (left panel). Statistically significant differences between the groups are denoted by asterisks (*p* < 0.01). **(E)** The panel provides a visual representation of the vibrations exhibited by a C3 whisker when encountering P220 texture as a wheel touches the tip of the whisker, moving at two different velocities (130.2 and 147.3 deg/sec). **(F)**. The influence of surface velocity on the SD of whisker vibration position and curvature for the whiskers in **(E)**. The lines represent the linear regression fit to the data. **(G)** The influence of surface velocity on the SD of whisker vibration position and curvature across all recorded whiskers. Statistically significant differences between the groups are denoted by asterisks. (*p* < 0.01). **(H)** The dependence of the normalized SD of both whisker vibration position and curvature on surface velocity varied according to the texture identity, measured as the slope of the linear regression **(F)**. **(I)** The panel provides a visual representation of the vibrations exhibited by a C3 whisker when encountering P220 texture as a wheel touches the whisker at three distances. **(J)** The influence of surface distance on the SD of whisker vibration position and curvature for the whiskers in **(I)**. The lines represent the linear regression fit to the data. **(K)** The influence of surface distance on the SD of whisker vibration position and curvature across all recorded whiskers (*p* < 0.01). **(L)** The dependence of the normalized SD of both whisker vibration position and curvature on surface distance was not dependent on whisker distance, measured as the slope of the linear regression **(J)**.

To replay whisker movements across different surfaces during receptive sensing in awake behaving rats, we covered the face of a rotating cylinder with several grades of sandpaper with varying degrees of coarseness and rotated the cylinder against the whiskers (Figure 1A). The cylinder face was placed so that the whiskers of the subject rats rested upon it (Figure 1A), and it was positioned to mimic rostral-caudal whisker movement during head movement. The head velocities associated with rat exploration were taken from Lottem and Azouz (Lottem and Azouz, 2009; Gugig et al., 2020). A DC motor was used to control the cylinder velocity to replicate the *median head movement velocity*. The 30 mm diameter wheel, powered by a DC motor (Farnell, Leeds, UK) was utilized in the experiment. Four surfaces with different coarseness grades were employed, ranging from coarse to fine-grained. The grain sizes of these surfaces, indicated in parentheses in microns, were as follows: P120 (125), P220 (68), P400 (35), and P800 (21). These grades were selected based on previous studies (Arabzadeh et al., 2005; Hipp et al., 2006; Morita et al., 2011). Whisker displacements transmitted to the receptors in the follicle were measured using a Mikrotron CoaXPress 4CXP camera, operating at 1600 frames per second and with a resolution of 4 Megapixels. The camera was positioned above the arena, providing an overhead view. The recorded movies were analyzed using the Janelia whisker tracker software (Clack et al., 2012). To calculate whisker curvature, which allows for the estimation of forces acting on the whisker follicle (Birdwell et al., 2007; Quist and Hartmann, 2012), we employed the method previously reported by Towal et al. (2011) (Birdwell et al., 2007). This analysis measured curvature at 10 points along the whisker, and the maximum local curvature per image was extracted.

### Data analysis

We established a trial structure to examine the influence of surface coarseness on whisker motion and resulting cortical neuronal responses. The cylinder rotated for 500 ms for each texture and remained still for 1500 ms. This sequence was repeated 75–150 times. We then aligned the whisker responses and the corresponding neuronal responses to the beginning of cylinder movement to generate PSTHs (Figures 1E, F).

The electrophysiological data was sampled at a frequency of 25KHz. The resulting spikes were stored with a temporal resolution of 100 μs. In parallel, whisker movements were recorded in a video format at a rate of 1600 frames per second. The two signals were aligned to establish the correlations between these two data streams. This was primarily accomplished by subsampling the spike timing information at 1 ms.

Statistical analysis was performed using a one-way analysis of variance (ANOVA) to determine the significance of differences among the measured parameters. In cases where significant differences were detected in the F ratio test (*P* < 0.05), Tukey’s multiple comparisons method was employed to identify specific pairs of measured parameters that exhibited significant differences from each other within a parameter group (*P* < < 0.01). The mean values are presented with the corresponding standard deviation (SD). Error bars in all figures represent the SD unless otherwise stated.

### Receiver operating characteristics analysis

We used signal detection theory [receiver operating characteristics (ROC) analysis (Green and Swets, 1974)] to compute the probability that an ideal observer could accurately determine the differences among the different textures based on neuronal activity. For each measured texture pair neuronal responses, an ROC curve was constructed as a two-dimensional plot of hit probability (*y*-axis) and a false alarm (*x*-axis) probability. Green and Swets (1974) demonstrated that the area under the ROC curve (AUC) corresponds to the performance expected of an ideal observer in a two-alternative, forced-choice paradigm, such as the one used in the present analysis. The ROC curve was calculated for a single neuron’s firing rate as a texture function. We then averaged all AUC values of all neurons and all texture pairs.

To transform raw data into a measure of discriminability, we analyzed the distributions of neuronal firing rates across trials. The firing rate (*Fr*) in trial *k* is the spike count = *Nsp*_*k*_ divided by *T*, trial duration in ms.


$$F\,r=\frac{N\,s\,p_{k}}{T}$$

The length *T* for the texture signal was set to *T* = 500 ms.

To assess the significance level of the AUC values we got from each neuron for all texture comparisons, we shuffled the firing rates of all trials among the various textures. We then calculated the ROC curves and AUC values for the shuffled data. We then averaged all AUC values of all neurons and all texture pairs. This process was repeated 500 times. The significance level, set at mean + 2SD (95%), was AUC = 0.53.

### Texture selectivity

A neuron responding to several textures shows a higher or lower firing rate for a particular texture, and this neuronal property is referred to as texture selectivity (Garion et al., 2014). An additional criterion for texture selectivity implemented was determining whether a specific texture had a significantly higher or lower firing rate (or any other parameter) than all other textures.

To calculate the texture selectivity of cortical neurons, we used the Selectivity Index (SI).


$$S\,I=M\,a\,x\,\left(P\,i\right)-\bar{\left(P\,j\right)}/M\,a\,x\,\left(P\,i\right)$$

*where P is the firing rates; i = preferred Texture; j = All Texture excluding the preferred texture; Max(Pi) = maximal firing rate; $\bar{\left(P\,j\right)}$ = the average firing rates across all textures*.

To quantify the statistical significance of texture selectivity, we first calculated the SI for several textures using the SI formula outlined above. Second, for each neuron, we have *n* × 75 trials, where *n* represents the number of textures, and 75 signifies the number of trials conducted for each texture. We randomly shuffle all trials across different textures to compute the SI. We iterate this process 500 times, calculating the average surrogate SI and SD afterward. We calculated the ‘mean + 3SD’ from this 500 SI data distribution. If the original SI surpassed the surrogate SI (mean + 3SD), this confirmed that the texture selectivity was not merely a product of chance.

### Surface coarseness impact on neuronal responses

Upon plotting the neuronal response characteristics corresponding to various textures, we discern a complex and interconnected relationship between these parameters, as depicted in Figures 1 through 6. To quantify these complex relationships, we divided the neural responses as a function of surface velocity and distance into four categories (Figure 6C lower panels):

1. Up-neurons presenting a significant monotonic increase.
2. Down–neurons presenting a significant monotonic decrease.
3. Complex–neurons exhibiting intricate changes in firing rate in response to wheel velocity and surface distance alterations.
4. No change–neurons that did not show any significant changes.

To categorize the diverse dependencies observed, we established specific empirical rules. These rules were strategically designed to classify these groups based on visually discernible characteristics distinctly. They were set to be both minimal and comprehensive enough to accurately divide the dependencies into their respective visually inspected groups. We discovered that, for categories 1 and 2, when at least 3 out of 4 neuronal responses to different textures (4 textures) displayed consistent and statistically significant ascending (upward) or descending (downward) trends in various aspects of their neuronal responses, it corresponded to the visually inspected dependencies.

## Results

In this study, we aimed to explore the transformation of whisker interactions with surfaces into neuronal activity in the cortex and how different stimulus configurations influence this process. To simulate the receptive sensing of whiskers encountering different surfaces, we utilized sandpapers with varying degrees of coarseness, which were selected from different grade levels (Lottem and Azouz, 2008). By rotating a cylinder covered with sandpaper, we replicated the receptive whisker sensing experience. The cylinder face was positioned perpendicular to the vibrissae, allowing them to rest upon it (Figure 1A). Figure 1B demonstrates how texture distance can affect whisker motion. Through this experiment, our goal was to understand better how the cortex processes and represents tactile information.

### The influence of stimulus configurations on tactile inputs

To quantitatively evaluate the impact of texture coarseness on whisker angle and curvature, we measured the position and curvature SD of each measured whisker vibration in response to all studied textures. The SD was calculated throughout the 500 ms stimulus duration. We then quantified the range of whisker vibration in response to the different textures. Our results showed a clear relationship between texture coarseness and whisker response characteristics, with coarser surfaces resulting in higher response SD values and finer surfaces resulting in lower response SD values (Gugig et al., 2020; as seen in Figure 1C for the recording in Figure 1B). These findings were consistent across all recordings for all whiskers (as depicted in Figure 1D; *n* = 20) and suggest that surface coarseness significantly impacts the amplitude of SSE changes (Wolfe et al., 2008; Gugig et al., 2020).

To explore these transformations further, we varied stimulus configurations by changing the surfaces’ velocity and distance. We used three velocities (130.2, 147.3, 169.5 deg/sec) and three distances from the whisker tip (Tip, 5 mm away from the tip, 10 mm away from the tip). An example of the influence of surface velocity on the whiskers’ signal is shown in Figure 1E for P220 and C3 whiskers. Quantification of these signals shows that an increase in wheel speed resulted in an increase and decrease of response SD movement and curvature values, respectively (Figure 1F). These findings were consistent across all recordings for all whiskers (as depicted in Figure 1G; *n* = 18; normalized to 147.3 deg/sec) and suggest that surface velocity significantly impacts the amplitude of SSE changes (Wolfe et al., 2008; Gugig et al., 2020). We used the linear regression fit calculated from Figure 1G for texture to examine whether these changes are texture-dependent. We used the slope of the regression to quantify this dependence. Figure 1H displays the normalized slope (normalized to P120) for all textures. Our findings reveal a direct correlation: as texture coarseness increases, the impact of velocity augmentation becomes more pronounced.

An example of the influence of surface distance on whisker vibrations is shown in Figure 1I for P220 and C3 whiskers. Quantifying these signals shows that getting closer to the pad increased response SD movement and curvature values (Figure 1J). These findings were consistent across all whisker recordings (Figure 1G; *n* = 18). We used the linear regression fit calculated from Figure 1K for each texture to examine whether these changes are texture-dependent. Figure 1L shows the normalized slope (normalized to P120) for all textures. Our study uncovered a significant pattern: the rougher the surface, the more pronounced the effect of distance increments. Together, these results strongly indicate that stimulus configurations exert a significant influence on tactile inputs to the whisker somatosensory system by altering the kinetic and kinematic parameters of whisker vibrations.

### The influence of stimulus configurations on texture discrimination and stability

**FIGURE 2:**
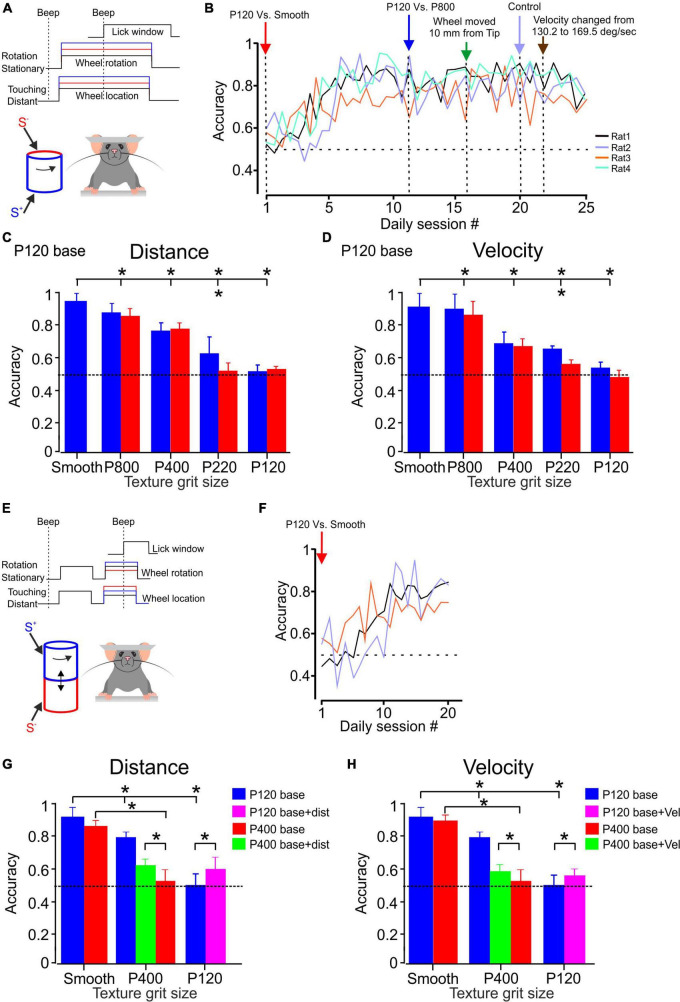
Texture discrimination and stability in awake head-fixed rats. **(A)** The whiskers contact a rotating cylinder coated with textured surfaces in the experimental design. The rats display licking behavior exclusively when they perceive a difference between the S^+^ and S^–^ stimuli, regardless of whether they are under control conditions or experiencing changes in stimulus configurations (indicated by the red and blue lines). Further details can be found in the methods section. **(B)** The learning curves for discrimination between P120 sandpaper and smooth surface are presented. The learning curve for four rats is shown for sandpaper discrimination. The panel demonstrates the temporal progression of the experiment. Once each animal achieved a certain level of discrimination (see text), we made changes such as introducing a different texture (P800, P400, P220, P120) as the S^–^ stimulus or altering the distance between the wheel and the pad, as well as the velocity of the wheel. **(C)** The psychometric curve for sandpaper texture discrimination. The average performance of four rats in P120 base discrimination is presented. The blue bars represent the SEM across daily blocks for texture discrimination when the surface made contact with the whisker at the tip. In contrast, the red bars represent the SEM when the surface is closer to the pad, positioned 10 mm farther from the tip. **(D)** Same as **(C)** for velocity. The control velocity was 147.3 deg/sec and was then adjusted to 169.5 deg/sec. **(E)** In the experimental design, the whiskers come into contact with a rotating cylinder covered with textured sandpaper. The rats exhibit licking behavior only when they detect a distinction between the S^+^ and S^–^ stimuli, regardless of whether the second stimulus presentation occurs under control conditions or when changes in stimulus configurations are introduced (as indicated by the red and blue lines). For more comprehensive information, please refer to the methods section. **(F)** The presented learning curves depict discrimination performance for the second paradigm involving P120 sandpaper and a smooth surface. Further details can be found in the methods section. **(G)** The psychometric curve for sandpaper texture discrimination. The graph illustrates the average performance of three and two rats in P120 (blue) and P400 (red) base discrimination tasks, respectively. In these tasks, the rats were presented with two stimuli. The green and pink bars represent the discrimination performance when the second stimulus was presented with the surface closer to the pad, positioned 10 mm farther from the tip. **(H)** Same as **(G)** for velocity. *Significantly different *p* < 0.01.

In the study, we investigated the impact of stimulus configurations on texture discrimination using a sandpaper discrimination task with rats. The experiment consisted of two primary measures: texture discrimination and stability. The training procedure involved six head-fixed rats, and the investigation followed a specific paradigm (Paradigm1; Figure 2A). Initially, the rats were trained to drink from a reward port to familiarize them with the task. After this initial training, a rough sandpaper (P120), denoted as S^+^, and a smooth plastic film, marked as S^–^, were presented side-by-side on a rotating wheel. During the experiment, the rats were required to keep their whiskers stationary. After a brief period, the wheel was moved to a position where the rat’s whiskers made contact with the rotating wheel. When the wheel began moving, the rat had the opportunity to lick a spout, which resulted in a water reward. The ability of the rat to successfully lick the spout indicated its capability to distinguish between the two textures (S^+^ and S^–^). Once the rats reached a stable level of discrimination, we conducted further tests by replacing the smooth plastic film (S^–^). This additional step aimed to assess the rats’ acuity or sensitivity to texture differences.

We calculated discrimination accuracy for each rat to evaluate the rats’ performance in the sandpaper discrimination task. Discrimination accuracy is the proportion of correct trials out of all trials conducted daily for a specific pair of textures, with a target of approximately 125–150 trials per day. By analyzing discrimination accuracy, we can assess the rats’ ability to distinguish between the rough sandpaper (S^+^) and other textures (S^–^) and determine if their discrimination skills improved. The accuracy measure provides insights into the rats’ learning progress and overall performance in the task. Figure 2B illustrates the performance of four rats in the discrimination task. The figure demonstrates that these rats gradually improved discrimination accuracy over time. They eventually reached a criterion performance level, defined as achieving a performance of at least 75% correct trials for two consecutive days. The data in Figure 2B suggests that these four rats took 5–10 days to attain the criterion performance level. This indicates they required varying training and practice before consistently performing above the specified performance threshold.

After the rats reached the discrimination criterion, we changed the stimulus configuration to examine the rats’ ability to distinguish between two textures despite changes in stimulus configurations. Specifically, we altered the S^–^ texture and measured the rats’ accuracy over several days to assess their performance under the new conditions. Once the discrimination performance became stable under the modified S^–^, we introduced additional factors related to the wheel distance and velocity. Figure 2B presents the results of these modifications and demonstrates how the wheel distance and velocity introduction affected the rats’ accuracy. It shows that different distances and velocities of the rotating wheel do not significantly impact the rats’ ability to discriminate between the textures (S^+^ and S^–^).

Figures 2C, D (blue bars) illustrate the performance of all four rats in different discrimination tasks. The results indicate that the rats achieved high accuracy in discriminating between specific pairs of sandpaper textures. All four rats exhibited high accuracy for the P120 vs. P800 discrimination task, with an average correct rate of 87%. This performance was significantly above the chance level, as indicated by a p-value of less than *p* < 1.3 × 10^–7^ based on a binomial exact test. Similarly, the rats demonstrated strong and significant discrimination abilities in the P120 vs. P400 and P120 vs. P220 tasks. However, when the test and base sandpapers were P120 in roughness, the rats performed at chance level (49–51% correct, *p* > 0.32).

Figures 2C, D showcase the rats’ performance in different discrimination tasks and highlight the impact of changing texture distance and velocity on discrimination accuracy, as represented by the red bars (Distance change–10 mm farther from the tip; velocity change–169.5 deg/sec). The results indicate that, except for the P120 vs. P220 discrimination task, changes in stimulus configuration, specifically texture distance and velocity, did not significantly influence discrimination performance. Thus, altering the distance and velocity of the textures presented to the rats did not substantially affect their ability to discriminate between the surfaces in most tested tasks. The rats’ discrimination performance remained consistent regardless of these changes in stimulus configuration.

After evaluating texture discrimination, we assessed texture stability (paradigm 2). Texture stability refers to maintaining a consistent and accurate representation of a texture despite variations in stimulus configurations. We introduced changes to the stimulus configurations to test for texture stability while keeping the surfaces constant. These changes included altering texture distance and velocity. By examining the rats’ performance in maintaining accurate texture representations across different stimulus configurations, we determined the extent to which the rats could maintain a stable perception of the texture.

In paradigm 2, the training procedure involved five head-fixed rats. The rats were presented consecutively with rough sandpaper (P120; *n* = 3; P400; *n* = 2), S^+^, and a smooth plastic film, S^–^, on a rotating wheel. Each trial began when the rotating wheel was placed so the rat’s whiskers touched it, which meant S^+^. Then, the wheel was retracted away from the rat’s whiskers. After a short while, the wheel was moved again, so the rat’s whiskers made contact with the wheel at a different location, representing S^–^. The rats were trained only to lick the spout when the wheel moved for the second time, showing their ability to tell the difference between the two textures (S^+^ and S^–^; Figure 2E). Successful licking of the spout resulted in a water reward, reinforcing the discrimination behavior. Once the rats reached a stable level of discrimination in this paradigm (P120-Smooth), we conducted further tests by replacing the smooth plastic film (S^–^) with different textures or the same textures at different distances or velocities. This additional step aimed to assess the rats’ texture stability. This enabled us to evaluate the rats’ ability to maintain a stable and accurate representation of the texture despite changes in the stimulus configuration.

Figure 2F illustrates the performance of three rats in the discrimination task. The figure demonstrates that these rats gradually improved discrimination accuracy over time in this paradigm. In comparison to the first paradigm, the results shown in Figure 2B indicate that the rats found this paradigm more challenging, requiring them approximately 20 days to reach the desired level of performance. After the rats reached the discrimination criterion, we changed the S^–^ to examine the rats’ ability to distinguish between two textures in the new paradigm. Figures 2G, H (P120 base–blue bars; P400 base–red bar) illustrate the performance of all rats in different discrimination tasks. The results indicate that the rats achieved high accuracy in discriminating between specific pairs of sandpaper textures. All four rats exhibited high accuracy for the P120 vs. smooth and P400 discrimination tasks, with an average correct rate of 79%. This performance was significantly above the chance level, as indicated by a p-value of less than *p* < 2.4 × 10^–6^ based on a binomial exact test. However, when the test and base sandpapers were P120 and P400 in roughness, the rats performed at chance level (47–53% correct, *p* > 0.41).

Once we attained consistent discrimination performance in the new experimental setup, we introduced further variables regarding the distance and velocity of the wheel. Therefore, “S^–^” signifies the wheel making contact for the second time, representing the same texture but at a distinct distance or velocity. The effects of these modifications are depicted in Figure 2G, showcasing how altering the wheel distance influences the accuracy of the rats’ performance. The graph reveals that varying distances of the rotating wheel affect the stability of texture perception for the base texture of P120 (indicated by the pink bar) and P400 (indicated by the green bar).

These findings hold also when the wheel velocity is altered, as shown in Figure 2H. The impact of these adjustments is illustrated in Figure 2G, providing insights into how changes in the wheel’s velocity impact the rats’ performance accuracy. The graph also reveals the influence of the velocity of the rotating wheel on the stability of texture perception for the base textures P120 (pink bar) and P400 (green bar).

Hence, with alterations in stimulus configurations for P120 and P400, the rats exhibited distinct texture perception, evident from their ability to discriminate between the same texture at different velocities. These findings indicate that modifying the distance and velocity of the presented surfaces notably affected the rats’ ability to maintain stability in most tested tasks. The rats’ discrimination performance varied considerably depending on these modifications in stimulus configuration.

### The influence of stimulus configurations on cortical neuronal responses

**FIGURE 3:**
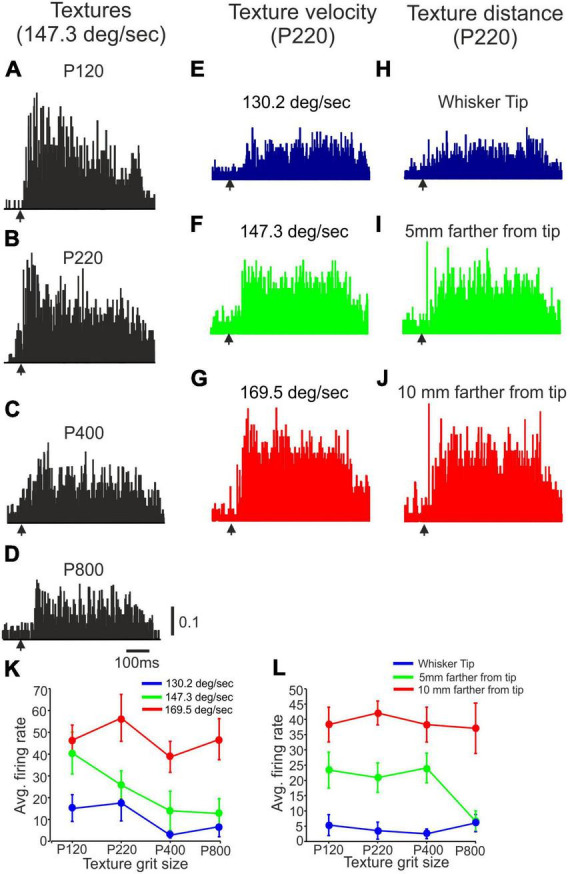
The impact of stimulus configuration on neuronal responses to textures. **(A–D)** Representative PSTHs showing neuronal responses to four surfaces (P120, P220, P400, P800) presented at 147.3 deg/sec at the whisker’s tip. **(E–G)** Representative PSTHs showing neuronal responses to P220 presented at three different velocities (130.2 deg/sec, 147.3 deg/sec, 169.5 deg/sec) at the whisker’s tip. **(H–J)** Representative PSTHs showing neuronal responses to P220 at 147.3 deg/sec were presented at three different distances (Tip, 5 mm farther from tip, 10 mm farther from tip). The vertical scale bar for PSTH shows the spike probability/msec bin. **(K)** The impact of surface velocity on the influence of surface coarseness on neuronal firing rates of the neuron in **(E–G)**. **(L)** The effect of surface distance on the influence of surface coarseness on neuronal firing rates of the neuron in **(H–J)**.

In this section, our objective is to examine the impact of different stimulus configurations on the firing rates of cortical neurons. To achieve this, we manipulated various aspects of the stimuli, including surface coarseness, distance, and velocity. By doing so, we can investigate the specific factors that influence the responses of neurons in the cortex. Figures 3A–J illustrate the responses of a representative single neuron to different combinations of surface coarseness, velocity, and distance. Figures 3A–D present the neuron’s responses to different surface coarseness levels (P120, P220, P400, P800; 147.3 deg/sec delivered at whisker tip). Furthermore, Figures 3E–G showcase the neuron’s responses to P220 at different velocities (130.2, 147.3, 169.5 deg/sec), while Figures 3H–J illustrate the responses to P220 at different distances (Whisker tip, 5 mm farther from tip, 10 mm farther from tip). These figures show how this particular neuron responds to variations in surface coarseness, velocity, and distance, shedding light on the specific factors that influence its activity and firing patterns.

To quantitatively analyze these changes, we plotted the neuronal firing rates for all possible combinations of stimulus configurations. Figures 3K, L demonstrate the effects of texture distance and velocity on the neuron’s response to different textures. The figures clearly illustrate that, for this particular neuron, surface distance and velocity play a crucial role in determining the dependence of firing rates on surface coarseness. Notably, these influences are observed to be dependent on the specific textures used, indicating that the characteristics of the textures interact with distance and velocity to shape the neuronal response. These findings highlight the intricate relationship between stimulus configurations and neuronal activity.

We categorized the neurons into four groups to analyze the impact of changes in wheel velocity and surface distance on neuronal firing rates. These groups allowed us to study the specific effects of these changes on neuronal response patterns. The first group, “Up,” exhibited a monotonic increase in firing rate with increased wheel velocity and decreased surface distance. The second group, labeled “Down,” showed a monotonic decrease in firing rate with the same changes. The third group, called “Complex,” displayed intricate changes in firing rate in response to wheel velocity and surface distance alterations. Lastly, the fourth group, termed “No change,” did not exhibit statistically significant changes in firing rate with the changes made.

**FIGURE 4:**
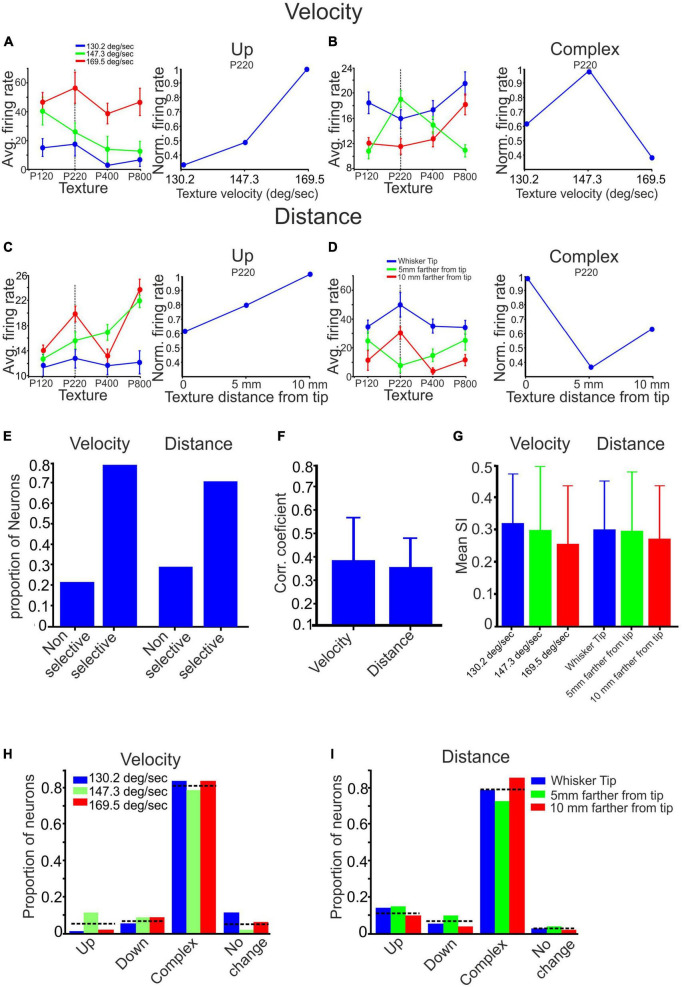
The impact of stimulus configuration on responses to textures. **(A–D)** The interplay between surface velocity and distance yielded complex effects. **(A,C)** When surface velocity increased, and surface distance decreased, there was an observed monotonic increase in firing rates for the P220 texture. **(B)** Increased surface velocity selectively enhanced the response to the P220 texture. **(D)** Decreased surface distance selectively reduced the response to the P220 texture. **(E)** The proportion of neurons displaying selective and non-selective responses to surface velocity and distance varied. **(F)** Changing surface velocity and distance impacted the correlation coefficient between texture-dependent firing rates in different stimulus configurations. **(G)** Stimulus configuration did not affect texture selectivity (see section Materials and methods). **(H,I)** Stimulus configuration did not impact the proportion of neurons showing texture selectivity.

Figures 4A, C provide examples of the “up” category, illustrating a monotonic increase in firing rates for P220 Stimulus as wheel velocity and distance from the tip increased. Figures 4B, D exemplify the “Complex” category, showcasing neurons that exhibit non-monotonic changes in firing rates in response to variations in wheel velocity and distance from the tip. These examples, along with the differential impact on different textures, emphasize the diverse and intricate nature of the effects of stimulus configuration on neuronal responses. The firing rates of neurons categorized based on stimulus configuration demonstrate how changes in wheel velocity and distance from the tip can lead to complex and varied effects on neuronal activity.

To evaluate the influence of surface distance and velocity on all neurons and textures, we consolidated these categories into two primary groups: selective and non-selective. The selective impact of stimulus configuration on a neuron was determined when there was a non-monotonic change in at least one of the responses to a specific texture (refer to Figures 4B, D). We then analyzed the proportion of neurons within each of these groups. Our findings indicate that selective influences of surface velocity were observed in 78% of the neurons. In comparison, non-selective influences were present in the remaining 22%, as illustrated in Figure 4E. Similarly, selective effects of surface distance were observed in 71% of the neurons. In comparison, non-selective influences were observed in the remaining 29%, as depicted in Figure 4E. These results indicate that most neurons exhibit selective responses to changes in surface distance and velocity. These findings demonstrate the intricate relationship between velocity, surface distance, and the firing patterns of the neuronal population, indicating a complex dependency in neuronal responses.

From Figures 4A–D, it is evident that altering the texture’s velocity or distance can significantly impact the relationship between firing rates and surface coarseness. We calculated the Pearson correlation coefficient (PCC) between the graphs associated with each neuron’s response to all textures under different conditions to examine these effects. For instance, in Figure 4A, we computed the PCC between the blue, green, and red graphs. A higher PCC value indicates a more substantial similarity between the conditions, as shown in Figure 4A. Conversely, a lower PCC value indicates a dissimilar dependence of firing rates on surface coarseness, as illustrated in Figure 4C. By calculating the mean values across all neurons and conditions, we found that the Pearson correlation coefficient (PCC) for all velocity conditions is 0.39 ± 0.19, as depicted in Figure 4F. Similarly, for all distance conditions, the PCC is 0.36 ± 0.12. These findings demonstrate that modifying the stimulus configuration affects the association between neuronal firing rates and surface coarseness.

As previously demonstrated, cortical neurons can be categorized into four groups based on their responses to different textures. These categories include the Up, Down, Complex, and No-change groups. Examples of these categories can be observed in all figures presented throughout the study. We used two approaches to investigate whether stimulus configuration changes affect texture classification. First, we calculated the texture selectivity index (SI) across all stimulus configurations. We found neither surface distance nor velocity significantly impacted SI (Figure 4G). To assess the impact of surface distance and velocity on all neurons and textures, we also investigated the changes in the proportions of neurons within each group. In the Up, Down, Complex, and No-change groups, the percentages of neurons that displayed changes in firing rate due to wheel velocity were 0.048, 0.072, 0.81, and 0.05, respectively, as depicted in Figure 4H. Similarly, for the surface distance groups categorized as Up, Down, Complex, and No-change, the percentages of neurons were 0.12, 0.07, 0.79, and 0.015, respectively, as illustrated in Figure 4I. These findings suggest that altering the stimulus configuration does not significantly impact the overall distribution of cortical neuron responses to surface coarseness.

### Discrimination and stability in cortical neuronal responses

**FIGURE 5:**
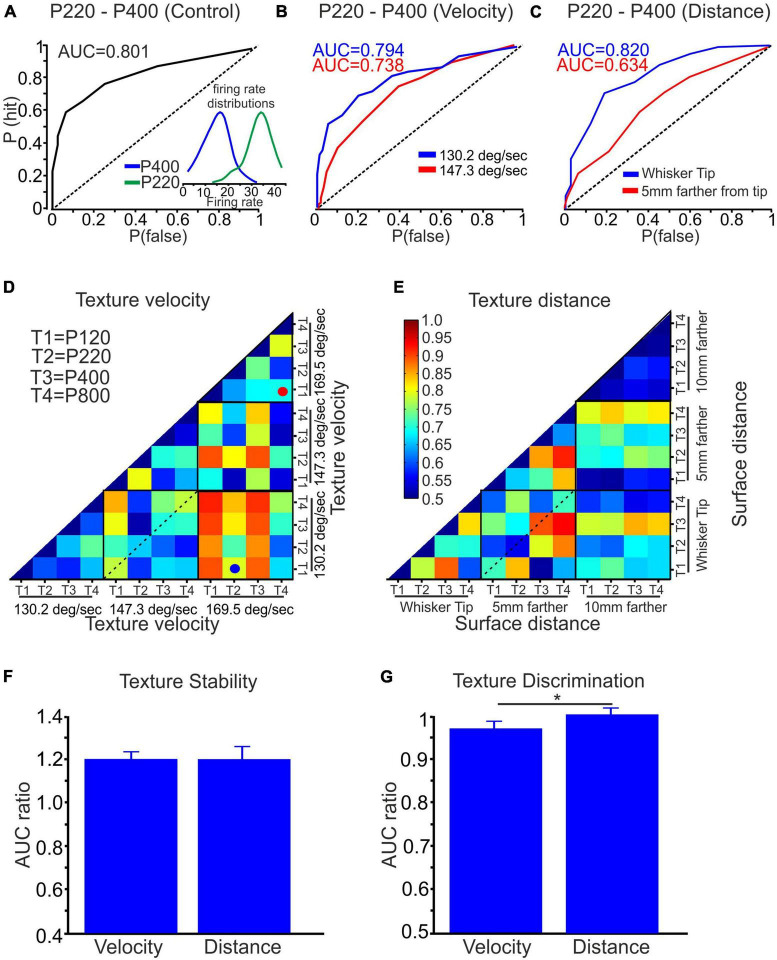
Quantification of texture stability and discrimination using ROC analysis. **(A)** The AUC, based on the ROC analysis, was calculated for textures P220 and P400, yielding an AUC value of 0.801. The inset depicts the firing rate distribution for surfaces P220 and P400. **(B)** The AUC of surfaces P220 and P400 was assessed while varying the wheel velocity from 130.2 to 147.3 deg/sec, examining its influence on the AUC. **(C)** Similarly, the effect of distance on the AUC was investigated by changing the distance from the whisker tip to 5 mm closer to the whisker pad for textures P220 and P400. **(D,E)** A color-coded matrix was generated to display the AUC values for the four textures at three surface velocities and distances, respectively. Each square in the matrix represents an average AUC value. **(F)** Influence of wheel velocity and distance on texture stability. **(G)** Effect of wheel velocity and distance on texture discrimination (*p < 0.05).

We utilized ideal observer analysis to assess the impact of stimulus configurations on neuronal discrimination and stability. This analysis allowed us to measure the discriminative power of each neuron quantitatively. Considering all the textures, we compared the firing rates across all trials for all stimulus configurations. To evaluate the discriminatory ability of each neuron, we employed the area under the receiver operating characteristic curve (AUC) measure. The AUC value quantitatively measures the neuron’s ability to discriminate between the textures, with higher values indicating better discrimination. We calculated the AUC for each neuron across all possible combinations of stimulus configurations. This analysis provided insight into how different stimulus configurations influenced neuronal discrimination and stability. Figure 5A presents an example of an ROC curve and the corresponding AUC for a neuron exposed to P220 and P400 textures. Figures 5B, C show ROC curves for two distinct neurons. These ROC curves depict the discrimination performance of the neurons when exposed to P220 and P400 textures at various velocities and distances. This analysis showed that increasing wheel velocity and distance from the whisker’s tip in these neurons decreased discrimination between the textures. This reduction in discrimination is reflected by the decrease in the AUC values, indicating a diminished ability of the neurons to differentiate between the surfaces under these conditions accurately.

In Figures 5D, E, we present the average AUC values across all neurons for all conditions. These figures utilize a color-coded pixel-based matrix representation, where each pixel corresponds to the average AUC value for a specific texture combination and stimulus configuration–for example, the red spot in Figure 5D signifies the average AUC of all neurons when comparing the responses to P120 (T1) and P800 (T4) textures at a velocity of 169.5 deg/sec. Similarly, the blue spot in Figure 5D indicates the average AUC of all neurons when comparing the responses to P220 (T2) textures at a velocity of 169.5 deg/sec and P120 (T1) textures at 130.2 deg/sec.

The matrices presented in Figures 5D, E provide two types of information: stability and texture discrimination. First, *Stability*: The diagonal values within each black square represent the average AUC values when comparing the responses to the same texture under different stimulus configurations (see dashed line in Figure 5D). These values reflect the stability of neuronal responses, indicating their ability to maintain consistent and accurate representations of a given texture despite changes in wheel velocity and surface distance. Second, *Texture Discrimination*: The non-diagonal values in the matrices represent the average AUC values when comparing the responses to different texture combinations under specific stimulus configurations. These values reflect the ability of neurons to discriminate between different textures despite variations in wheel velocity and surface distance. By analyzing stability and texture discrimination, these matrices provide a comprehensive understanding of how wheel velocity and surface distance variations impact neuronal responses.

To assess the statistical significance of coding stability and discrimination, we performed surrogate analysis by shuffling the firing rates of 75–150 trials between every two textures 500 times. We calculated the AUC for each shuffling and derived the mean plus three standard deviations (mean+3SD) from the resulting AUC data distribution. We then calculated the AUC ratio by dividing the original AUC value by the AUC value of the shuffled data. The analysis revealed that for coding stability, the AUC ratio was 1.2 for wheel velocity and surface distance changes. This analysis indicates that changing the stimulus configuration reduced coding stability, as demonstrated in Figure 5F. In contrast, the AUC ratio for discrimination was approximately 1, suggesting that wheel velocity and surface distance did not significantly affect texture discrimination, as shown in Figure 5G. These results provide evidence that changing stimulus configuration adversely impacts coding stability while having minimal effect on texture discrimination.

### Discrimination and stability in response to single- vs. multi-whiskers stimulation

Rats encounter objects in their environment with a variable number of whiskers, and these multiple tactile channels serve as essential tools for tactile perception, spatial awareness, and navigation in their natural habitats. To examine the influence of single whisker vs. multiple whisker stimulation on the transmission of tactile information, we recorded neuronal responses to single whisker texture stimulation (as was done previously), and for the same neurons, we recorded the responses to the same stimuli by multiple whiskers (*n* = 30; We used a limited number of conditions: P220 and P400, at the whisker tip and 5 mm away from the tip and at two velocities, 147.3 and 169.5 deg/sec.

**FIGURE 6:**
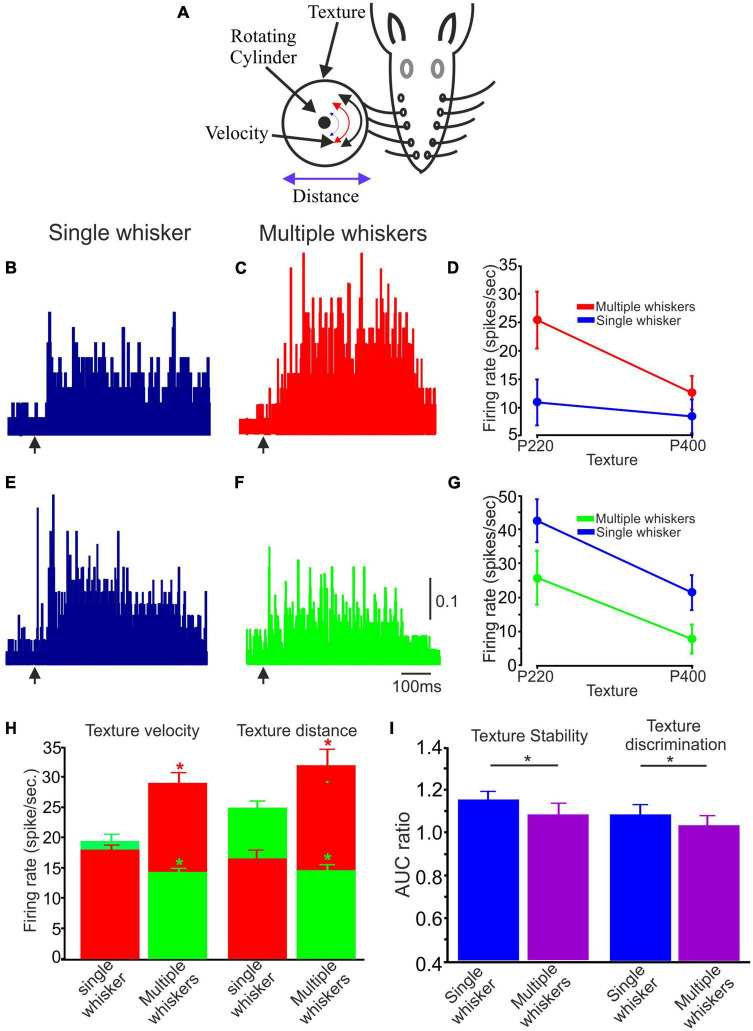
The impact of Single vs. multiple whiskers on texture stability and discrimination. **(A)** Experimental design. Similar to Figure 1A with numerous whiskers. **(B,C,E,F)** Two example PSTHs of single and multiple whiskers, respectively. The arrow indicates the starting point of the stimulus. **(B,C)** One illustrative case is a neuron that exhibited increased firing rates when presented with multiple whisker stimuli. **(E,F)** One exemplary case is a neuron that exhibited decreased firing rates when presented with multiple whisker stimuli. The vertical scale bar for PSTH shows the spike probability/msec bin. **(D)** Average firing rate for 75 trials associated with textures P220 and P400 in single and multiple whiskers for the neuron in **(B,C)**. **(G)** Average firing rate for 75 trials related to textures P220 and P400 in single and multiple whiskers for the neuron in **(E,F)**. **(G)** Influence of multiple whiskers on neuronal firing rate in the two stimulus configurations (surface velocity and distance). The two colors in the histograms represent the two populations of multiple whiskers compared to a single whisker (Red –increased firing rates; Green–decreased firing rates. **(H)** The figure shows that multiple whisker stimulation significantly changes cortical neuron firing rates compared to single whisker stimulation. The figure presents data from two subpopulations of neurons: those with decreased firing rates (green bars) and those with increased firing rates (red bars) in response to multiple whisker stimulation. The decreased firing rate group was tested under velocity and distance change conditions. **(I)** Influence of multiple whiskers on texture stability and discrimination. AUC ratio of coding stability in single vs. multiple whiskers (*p < 0.05).

When comparing single- to multi-whisker stimulation, two distinct populations of neuronal responses were observed: Neuronal firing rates were higher in response to multiple-whisker stimulation than single-whisker stimulation (Figures 6B, C); Neuronal firing rates were lower in response to multiple-whisker stimulation than single whisker stimulation (Figures 6E, F). Quantification of these two neurons is shown in Figures 6D, G, respectively. We then quantified the proportion of neurons in each population; 0.55 were associated with the higher firing rate group, and 0.45 were associated with the lower firing rate group. Figure 6H (green bars) presents examples or averaged data from a specific subpopulation of neurons characterized by decreased firing rate in response to multiple whiskers. These neurons were divided into two populations, where the first population was tested under velocity change conditions and the second population under distance change conditions. Conversely, the figure also includes examples or averaged data from another distinct population of neurons characterized by an increased firing rate (red bars). This figure effectively demonstrates that when multiple whiskers are stimulated, cortical neurons exhibit notable increases and decreases in firing rates compared to situations involving the stimulation of a single whisker.

Subsequently, we investigated whether the stimulation of multiple whiskers influences texture stability and discrimination in the two groups (increase and decrease in firing rates). Figure 6I provides a graphical representation of the results, demonstrating that the AUC ratio for multiple whisker stimulation was significantly lower and approached a value of one for both stability and discrimination, in contrast to the single whisker condition. This finding suggests that activating multiple whiskers contributes to preserving coding stability and enhancing texture discrimination capabilities.

## Discussion

In this study, we aimed to investigate the transformation of whisker interactions with different surfaces into cortical neuronal activity and how various stimulus configurations impact this process. To replicate the receptive whisker sensing experience, we used sandpaper with varying coarseness levels and rotated a cylinder covered with sandpaper, allowing the vibrissae to rest upon it. The primary goal was to gain insights into how the cortex processes and represents tactile information derived from whisker interactions with different surfaces and the impact of this neuronal activity on perceptual discrimination and stability.

We quantitatively evaluated how texture coarseness influences the intensity of whisker angle and curvature vibrations, as measured by their SD, in response to different textures. Analyzing each whisker’s position and curvature variability, we discovered that coarser surfaces induced more significant response variability, whereas finer surfaces led to reduced response variability (Gugig et al., 2020). In our study, we also explored the impact of stimulus configurations for the first time. By adjusting the surface velocity and distance, we discovered that increasing the surface velocity intensified the response of angles and reduced the intensity of whisker curvature. Conversely, getting closer to the pad amplified the response intensity of angles and curvatures (Figure 1). It is important to note that the stimulus configuration’s impact depended on the surface’s texture.

We investigated two distinct parameters to analyze whisker vibrations in response to the presented textures: the SD of whisker position and curvature. Based on prior research (Birdwell et al., 2007; Towal et al., 2011; Quist and Hartmann, 2012), these metrics provide insights into the forces exerted on the whisker follicle, potentially reflecting the occurrence of stick and slip events that influence neuronal discharge probability (Isett et al., 2018; Gugig et al., 2020). Furthermore, whisker curvature, which significantly influences information transmission along the whisker to mechanoreceptors in the follicle by altering the forces and moments at the vibrissal base (Birdwell et al., 2007), was a proxy for changes in bending moment (O’Connor et al., 2010). Our findings indicate that whisker-pole contacts induced substantial whisker bending, which partially correlated with the whisker angle (Campagner et al., 2016) and elicited robust spiking activity.

Next, we investigated the impact of different stimulus configurations on firing patterns and activity of cortical neurons. We manipulated surface coarseness, distance, and velocity to explore the factors influencing neuron responses. Quantitative analysis of firing rates for all possible stimulus combinations revealed that surface distance and velocity play a crucial role in determining the dependence of firing rates on surface coarseness (Figures 3, 4). The influences of stimulus configurations on neuronal responses are observed to be dependent on specific textures, suggesting an intricate interaction between texture characteristics, distance, and velocity. These relationships may be attributed to the fact that most cortical neurons exhibit selectivity to whisker velocity and distance, and this selectivity is surface coarseness dependent. A notable finding from the current study is that stimulus configuration profoundly affects the relationship between firing rates and surface coarseness, as supported by the correlation (Figure 4F). Alterations in stimulus configuration resulted in significant changes in the relationship between surface coarseness and firing rates (Figures 4A–D). However, our findings indicate that altering the stimulus configuration does not significantly impact the overall distribution of cortical neuron responses, showing selectivity to surface coarseness (Figures 4G–I).

Using ideal observer analysis, we evaluated the influence of stimulus configurations on neuronal discrimination and stability. While the firing rates of neurons showed complex and variable responses to changes in wheel velocity and surface distance, altering the stimulus configuration reduced coding stability while having minimal impact on texture discrimination.

Finally, the study investigated the impact of single-whisker and multiple-whisker stimulation on cortical neuronal responses. It revealed two distinct populations of neurons, one showing higher firing rates with multiple-whisker stimulation and the other with lower firing rates. Ultimately, the results demonstrated that multiple-whisker stimulation enhanced neuronal texture discrimination capabilities and preserved coding stability compared to single-whisker stimulation.

To examine the behavioral implications of neuronal sensitivity, we engaged in sandpaper discrimination tasks to unravel the interplay between evolving stimulus configurations, cortical neuronal responses, and behavioral precision. These tasks taught rats to differentiate between rough and smooth sandpapers under varied conditions. The rats demonstrated a progressive enhancement in discrimination accuracy throughout training, ultimately achieving a consistent level of discrimination performance. Our findings indicated that manipulating surface distance and velocity exerted limited influence on the rats’ capability to distinguish between textures in most tasks, underscoring a uniform discrimination performance unaffected by these adjustments in stimulus configuration.

However, it became evident that modifying the distance and velocity of presented textures significantly impacted the rats’ capacity to maintain constancy in most tasks. As a result, considerable fluctuations in discrimination performance arose depending on these modifications to stimulus configuration. In conclusion, this study emphasizes the significance of stimulus configurations in modulating the processing of tactile information in the cortex. It reveals the intricate relationship between various stimulus properties and neuronal activity, providing valuable insights into the underlying mechanisms of sensory perception.

### Roughness constancy

When examining mechanisms for perceiving surface roughness, distinguishing between perceived roughness and roughness constancy is crucial. Roughness constancy, a unique aspect of perception, maintains consistent roughness ratings for specific surfaces despite variations in scanning conditions. Research, mostly involving primates, including humans, highlights tactile texture constancy driven by the nervous system’s ability to reduce susceptibility to environmental changes (Taylor-Clarke et al., 2004). Studies indicate that tactile texture perception remains primarily unaffected by an applied force (Lederman and Taylor, 1972; Lederman, 1981) or scanning speed (Lederman, 1974; Meftah et al., 2000; Boundy-Singer et al., 2017), dependent on active or passive scanning modes influencing roughness perception (Yoshioka et al., 2011). This constancy persists despite afferent fiber responses influenced by factors like scanning speed (Goodwin and Morley, 1987b; Phillips et al., 1992; DiCarlo and Johnson, 1999; Weber et al., 2013) and force (Goodwin and Morley, 1987a; Phillips et al., 1992; Saal et al., 2018). Establishing perceptual constancy involves integrating proprioceptive inputs from muscle afferents, joint receptors (Bosco and Poppele, 2001; Chapman and Beauchamp, 2006), cutaneous receptors (Hulliger et al., 1979; Burke et al., 1988; Johnson, 2004), and cortical regions containing exteroceptive and proprioceptive information (Vallbo and Johansson, 1984; Patel et al., 2006; Lieber and Bensmaia, 2020).

Roughness constancy research in rodents’ whisker somatosensory system is still in its early stages. Only two studies have investigated perceptual constancy, one focusing on object location and the other on texture discrimination. In the first study, Saraf-Sinik et al. (2015) examined the influence of perturbing wind on object localization tasks in awake-behaving rats. They show that the rats adapted their motor-sensory whisking strategies to safeguard the effectiveness of sensory encoding for object location, even when faced with external perturbations. In the second study, Zuo et al. (2011) trained rats to differentiate between grooved textures using their whiskers. After trimming some of the whiskers, the rats adjusted their whisking behavior, suggesting an adaptive response to compensate for lost sensory input underscores rats’ utilization of a flexible, “information-seeking motor strategy” over rigid motor programs. These studies suggest that motor and sensory variables operate in tandem through closed-loop mechanisms, encompassing precise motor-object-sensory conversions and sensory-motor pathways. These sensorimotor loops enable perceptual constancy, enabling the nervous system to accommodate alterations in subject-object interactions.

Our findings indicate that receptive sensing is insufficient to uphold perceptual constancy in both neuronal activity and texture discrimination tasks. As supported by the existing literature (as mentioned earlier), our viewpoint aligns with the notion that achieving perceptual constancy within the whisker somatosensory system necessitates active sensing. This dynamic sensing approach entails employing tailored motor-sensory whisking strategies to ensure the optimal efficacy of sensory encoding, particularly for preserving accurate object characteristics. Continued research is essential to unveil the intricate mechanisms driving perceptual constancy and the cognitive processes employed by the brain to accomplish this feat. By exploring these mechanisms, we can attain a more profound comprehension of the fundamentals of sensory perception, thereby enhancing our understanding of how the brain effectively integrates and processes information. This endeavor will allow us to grasp how the brain maintains consistent perceptions even when faced with fluctuations in sensory inputs, contributing to the broader knowledge of sensory cognition.

### Texture discrimination

We employed a receptive sensing approach to investigate how whisker vibrations translate into cortical neuronal activity, keeping the whiskers stationary while moving the surfaces. Rats actively use their whiskers to sweep across surfaces, enabling them to locate and distinguish objects in their sensory environment (Welker, 1964; Carvell and Simons, 1990; Sachdev et al., 2001; Bermejo et al., 2002; Berg and Kleinfeld, 2003; Knutsen et al., 2005). This active whisking behavior is often accompanied by head and body movements (Carvell and Simons, 1995; Brecht et al., 1997; Mitchinson et al., 2007; Ritt et al., 2008; Towal and Hartmann, 2008). However, rodents also utilize receptive whisker movements, relying on body and head movements to initiate whisker motion. In such cases, they maintain contact with walls and surfaces while running.

The behavioral paradigms used to study texture discrimination significantly impact how rats utilize their whiskers to sense the tactile environment and the neuronal processing involved in this task. Head-fixed animals can only sense surfaces by whisking against them. Nevertheless, a quantitative examination regarding the influence of whisking strategies on texture discrimination under these conditions has yet to be published. The stable conditions, wherein surfaces are consistently located, may affect whisking strategies and simplify perception and discrimination (Wolfe et al., 2008; Jadhav and Feldman, 2010). In contrast, free-behaving animals develop a purposeful whisking strategy for interactions between their whiskers and surfaces, seeking information to perceive and discriminate different textures. Thus, whisking behavior is primarily associated with gathering tactile information, while discrimination performance seems more closely linked to the specifics of whisker-surface interactions (Zuo et al., 2011; Zuo and Diamond, 2019a). However, these studies also distance the surfaces from the animals to avoid using the microvibrissae while extending and palpating their macrovibrissae against the surfaces. Recent findings show that free-behaving rats can discriminate fine tactile patterns while running without whisking (Kerekes et al., 2017).

Furthermore, how rats utilize their whiskers to perceive the tactile environment and the neural processing involved in this task is significantly molded by the behavioral paradigms employed in the exploration of texture discrimination. These paradigms also undoubtedly play a role in determining whether the animals need to use behavioral and neural mechanisms to uphold perceptual constancy. Hence, our behavioral experiments reveal an intriguing observation: alterations in stimulus configurations do not impact the rats’ capacity to differentiate between textures. This finding prompts us to formulate a hypothesis suggesting that the rats might not necessarily require an intricate representation of surface coarseness within this particular behavioral paradigm. Instead, they could focus on conducting a comparative analysis between two surfaces to detect any discernible changes (Waiblinger et al., 2015).

This concept may resonate in several studies where the animal is presented with two surfaces nearby or in succession. For instance, in such experimental setups, the animal’s ability to discriminate between textures might rely on something other than maintaining a detailed representation of surface coarseness. Instead, the emphasis could lie on the animal’s capacity to conduct a comparative analysis of the two surfaces, whether presented side by side (Morita et al., 2011; Kerekes et al., 2017) or one after the other (Waiblinger et al., 2015). This suggests that the ability to detect changes between textures could be a fundamental aspect of the rat’s tactile perception across various experimental contexts.

The second behavioral paradigm introduces a time delay between initial and subsequent stimuli, crucial for situations where required information is spread over time without immediate cues. Decision-making in these cases involves an accumulative mechanism beyond the somatosensory cortex’s faster time constants. While vS1 excels at processing immediate stimuli, it faces limitations for tasks requiring gradual information integration. The brain adapts by engaging regions suited for prolonged accumulation and informed decision-making (von Heimendahl et al., 2007; Zuo et al., 2011; Estebanez et al., 2012; Fassihi et al., 2017, 2020; Zuo and Diamond, 2019a,b).

## Data availability statement

The raw data supporting the conclusions of this article will be made available by the authors, without undue reservation.

## Ethics statement

The animal study was approved by the Ben-Gurion University of the Negev. The study was conducted in accordance with the local legislation and institutional requirements.

## Author contributions

HS: Data curation, Formal analysis, Investigation, Software, Writing – original draft. RA: Conceptualization, Data curation, Formal analysis, Funding acquisition, Investigation, Software, Supervision, Writing – original draft, Writing – review and editing.

## Footnotes

1
https://github.com/adredish/MClust-Spike-Sorting-Toolbox
